# Regorafenib in Refractory Metastatic Colorectal Cancer: A Multi-Center Retrospective Study

**DOI:** 10.3389/fonc.2022.838870

**Published:** 2022-03-30

**Authors:** Donghao Xu, Yu Liu, Wentao Tang, Lingsha Xu, Tianyu Liu, Yudong Jiang, Shizhao Zhou, Xiaorui Qin, Jisheng Li, Jiemin Zhao, Lechi Ye, Wenju Chang, Jianmin Xu

**Affiliations:** ^1^ Department of General Surgery, Zhongshan Hospital, Fudan University, Shanghai, China; ^2^ Department of Surgery, The First Affiliated Hospital of Wenzhou Medical University, Wenzhou, China; ^3^ Department of Medical Oncology, Qilu Hospital, Cheeloo College of Medicine, Shandong University, Jinan, China; ^4^ Department of Oncology, The Third Affiliated Hospital of Soochow University, Changzhou, Jiangsu, China; ^5^ Department of Colorectal and Anal Surgery, The First Affiliated Hospital of Wenzhou Medical University, Wenzhou, China; ^6^ Shanghai Engineering Research Center of Colorectal Cancer Minimally Invasive Technology, Shanghai, China

**Keywords:** colorectal cancer, salvage treatment, regorafenib, chemotherapy, immune therapy

## Abstract

**Background:**

Regorafenib improves progression-free survival (PFS) and overall survival (OS) in patients with refractory metastatic colorectal cancer (mCRC). Here, we report the treatment patterns of regorafenib in the third- or late-line setting for mCRC in four centers in China.

**Patients and Methods:**

Patients with refractory mCRC in four centers in China administered regorafenib from February 1, 2018 to June 31, 2021 were enrolled. Patients were grouped into 3 cohorts, namely, the monotherapy (regorafenib alone), chemo (regorafenib plus chemotherapy), and immune [regorafenib plus anti-PD1 (programmed cell death 1) antibodies] groups. Demographic, clinical, survival and safety data were retrospectively analyzed.

**Results:**

A total of 177 patients were included in this study. Of them, 116 (65.5%) were treated with regorafenib alone, while 28 (15.9%) and 33 (18.6%) were administered regorafenib plus chemotherapy and anti-PD1 antibodies, respectively. The median followed-up time was 9.2 months. The disease control rate (DCR) was 40.7%. The median PFS (mPFS) was 2.43 months and the median OS (mOS) was 12.2 months. The immune group had longer median PFS (3.5 m vs. 2.2 m, p = 0.043) compared with the monotherapy group. Patients administered regorafenib plus chemotherapy had longer median OS (15.9 m vs. 8.4 m, p = 0.032) compared with the monotherapy group. Patients who began regorafenib treatment at 120 mg had longer median PFS and OS compared with those who began at 80 mg (PFS: 3.7 m vs. 2.0 m; p <0.001; OS: 13.4 m vs. 10.2 m; p = 0.005). Patients with a final dose of 120 mg had longer median PFS and OS compared with the 80 mg or less group (PFS: 5.0 m vs. 2.3 m; p = 0.045; OS: UR (unreach) vs. 10.9 m; p = 0.003). There were 87.0% (154/177) patients who experienced AEs. Three groups had similar rates of AEs (86.2% vs. 89.3% vs. 87.9%; p = 0.89).

**Conclusion:**

Patients administered regorafenib alone or regorafenib in combination with other agents were relieved to some extent, with a disease control rate of 40.7%. Regorafenib plus anti-PD1 antibodies showed better PFS, while regorafenib plus chemotherapy had the most benefit in OS. There was no significant difference among three groups in terms of AEs.

## Introduction

Colorectal cancer (CRC) is one of the most common types of cancer diagnosed worldwide (1), with at least 50% of patients developing metastases (2). The treatment of metastatic CRC (mCRC) has progressed in recent years, especially in the field of immunotherapy. Patients with dMMR (deficient mismatch repair)/MSI-H (microsatellite instability) tumors are potentially responsive to the PD-1 blockades, but these only account for 5% of all mCRC patients (3, 4). For first- and second-line treatments, biologics such as cetuximab (anti-EGFR) and bevacizumab (anti-VEGF) have significantly increased progression-free survival (PFS) and overall survival (OS) (5, 6). In particular, patients with left-sided mCRC had better PFS with anti-EGFR therapy compared with those with right-sided CRC, even among patients with RAS and BRAF wild-type (7, 8). However, in third- and later-line settings, there are few options, namely, regorafenib (9–13), TAS-102 (14, 15), and fruquintinib (16), with limited efficacy.

Regorafenib is an orally available, small-molecule multikinase inhibitor that targets signaling pathways implicated in tumor angiogenesis, oncogenesis, and the tumor microenvironment (9). Two international randomized Phase III trials (CORRECT and CONCUR) had shown that regorafenib could improve OS and PFS as salvage treatment compared with placebo group (10, 11). However, the efficacy of regorafenib monotherapy was limited with median PFS times ranging from 1.9 months to 3.2 months (10–13). Therefore, several trials have explored the efficacy of regorafenib in combination with immunotherapy (17–21). Indeed, the superiority of combined treatments has been indicated in some small-sample phase II trials and retrospective studies. Especially the REGONIVO trial (17), a combination of regorafenib and nivolumab (an anti-PD1 antibody), showed promising efficacy (ORR (objective response rate), 33%; mPFS, 7.9 months) in 24 refractory MSS (microsatellite stable) mCRC patients. However, the promising result of the REGONIVO trial failed to be replicated by other trials, including a phase IIb trial (REGOMUNE) (18), a phase Ib/II clinical trial (REGOTORI) and some retrospective studies (19–21). Furthermore, some retrospective studies indicated regorafenib plus chemotherapy improves PFS and OS (22–24). Nonetheless, most studies were small-sample and single-arm, and trials comparing these combined strategies are unavailable.

Here, we report large-scale data of regorafenib alone or combined with other therapies in four colorectal cancer centers in China. The aim of this study was to compare the efficacy and safety of regorafenib monotherapy, regorafenib plus chemotherapy, and regorafenib plus immunotherapy.

## Materials and Methods

### Study Design and Patients

This retrospective study was conducted in four comprehensive cancer centers in China. The demographic, clinical and survival data of all patients were retrospectively collected through electronic medical records.

Patients diagnosed with refractory mCRC and administered regorafenib from February 1, 2018 to June 31, 2021 were enrolled in this study. Inclusion criteria were: (1) histology-confirmed metastatic CRC (mCRC); (2) disease progression on standard therapy with at least two lines of chemotherapy, namely, fluorouracil, oxaliplatin, and irinotecan with or without biologics such as bevacizumab and cetuximab; (3) regorafenib administered as salvage treatment; and (4) available clinical data. Exclusion criteria were: (1) lack of follow-up data; (2) regorafenib administration as second-line treatment; (3) regorafenib administered in combination with HAIC; and (4) regorafenib administration stopped after less than two cycles.

Patients were grouped into three study cohorts according to the treatment received: (1) regorafenib alone; (2) regorafenib in combination with chemotherapy; (3) regorafenib in combination with anti-PD-1 antibodies.

Treatment evaluation was based on the Response Evaluation Criteria in Solid Tumor (RECIST) version 1.1 (25). The study was approved by the ethics committee of each institute, and performed according to Good Clinical Practice guidelines and the Declaration of Helsinki. Written informed consent was obtained from all participants.

### Multi-Disciplinary Team and Follow-Up

Multi-disciplinary team (MDT) discussion were performed weekly and the team included physicians from colorectal surgery department, liver surgery department, oncology department, radiation department, radiotherapy department, intervention treatment department, medical imaging department and thoracic surgery department. After the discussion of each CRLM patient, a consensus was reached on the treatment plan or further diagnostic work-up (26).

The general practice for surveillance included physical examination, serum CEA testing, CT of the chest, abdomen with intravenous contrast and MRI of abdomen, pelvis with intravenous contrast. These were performed every 2–4 cycles of regorafenib administration.

### Variables

The variables examined included sex, age, primary tumor site, metastatic tumor site, RAS and BRAF gene statuses, MMR (mismatch repair) status, primary tumor resection, treatment line, previous use of biologics, and initial and final dosages of regorafenib. RAS/BRAF mutations were detected with a KRAS/NRAS/BRAF Mutations Detection Kit, and MMR status was determined by immunohistochemistry (IHC).

### Outcomes

PFS, OS, disease control rate (DCR) and adverse events (AEs) were assessed in each cohort. PFS was calculated from the date of regorafenib administration to the first observation of disease progression or death. OS was defined as the time from regorafenib administration to death. DCR was determined as the proportion of patients who achieved complete response, partial response or stable disease assessed at least 6 weeks after drug administration. AEs included any grade symptomatic or hematological events, and were evaluated using the National Cancer Institute Common Terminology Criteria for Adverse Events (NCI-CTCAE V5.0).

### Statistical Analysis

Continuous variables were compared by the student’s t-test or the Mann–Whitney U test. Categorical variables were compared by the Chi-square test or Fisher’s exact test. Kaplan–Meier curve analysis and the log-rank test were used to assess PFS and OS. Death and disease progression were treated as events in the analysis. Hazard ratios (HRs) and 95% CIs (confidence intervals) were estimated using Cox regression models and assessed by the Wald test. Association factors with PFS and OS were evaluated by univariable and multivariable Cox regression analyses. All statistical analyses were performed with R version 4.1.1 (R Project). All tests were 2-sided, and P <0.05 was considered statistically significant.

## Results

### Patient Characteristics

**Figure 1:**
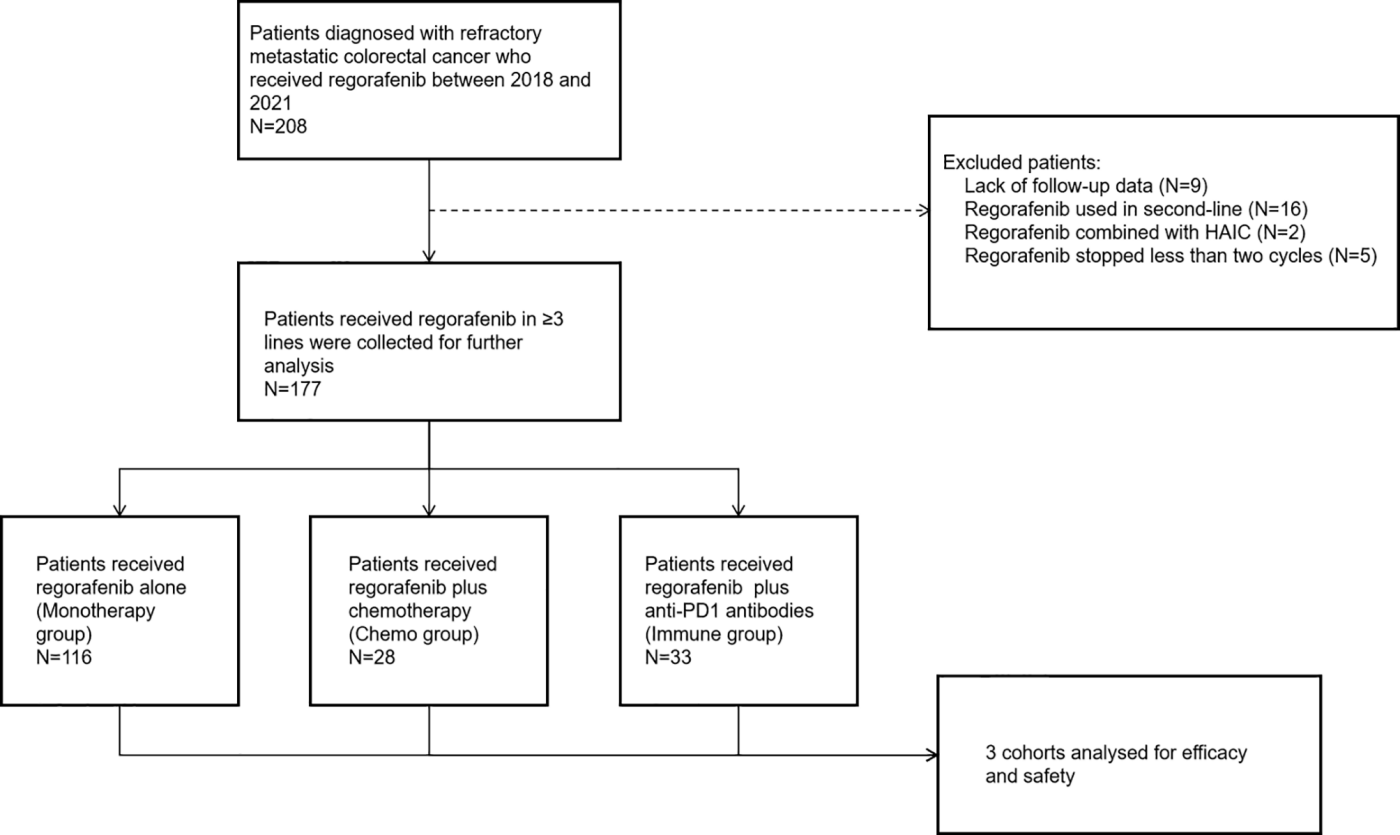
The profile of the present study.

A total of 208 consecutive patients diagnosed with refractory mCRC administered regorafenib from February 1, 2018 to June 31, 2021 were enrolled. Patients were excluded for lack of follow-up data (n = 9), regorafenib administration as second-line treatment (n = 16), regorafenib administered in combination with HAIC (n = 2), and regorafenib administration stopped after less than two cycles (6 weeks) (n = 5). In total, 177 patients who met the eligibility criteria were further analyzed (**Figure 1**).

In the 177 patients, median follow-up time was 9.2 months. The median age was 60 years (range: 32–82 years), and 102 (57.6%) patients were men. There were 82 (46.3%), 36 (20.3%) and 59 (33.3%) patients with left-sided, right-sided, and rectal cancers. In total, 88 (49.7%) patients were RAS mutant, 5 (2.8%) patient was BRAF mutant, and 84 (47.5%) patients were of RAS/BRAF wild type. Meanwhile, 174 (98.3%) and 3 (1.7%) patients had pMMR (proficient mismatch repair) and dMMR (deficient mismatch repair) statuses, respectively. Liver and lung were the most common sites of metastasis with 121 (68.4%) patients and 71 (40.1%) patients involved, respectively. In total, 65 (36.7%) and 138 (78%) patients were administered cetuximab and bevacizumab as prior systemic therapy, respectively. In total, 114 (64.4%) and 63 (35.6%) patients received regorafenib as third-line and later-line treatment, respectively. The most common starting dose of regorafenib was 80 mg (94 [53.1%]) (**Table 1**).

**Table 1:** Baseline demographic and clinical characteristics of 177 mCRC patients.

Characteristics	All Patients (n = 177)	
	n	%
**Sex**		
Men	102	57.6
Women	75	42.4
**Age(years)**		
Median (range)	60 (32–82)	
<65	121	68.4
≥65	56	31.6
Site of primary disease*		
Left-sided	82	46.3
Right-sided	36	20.3
Rectum	59	33.3
**Gene mutation status**		
RAS/BRAF wild-type	84	47.5
RAS mutant	88	49.7
BRAF mutant	5	2.8
**MMR status**		
pMMR	174	98.3
dMMR	3	1.7
**Site of metastases^†^**		
Lung limited	35	19.8
Liver limited	77	43.5
Liver and lung	36	20.3
Other	29	16.4
**Primary tumor resection**		
Yes	154	87
No	23	13
**Metastasectomy**		
Liver	26	14.7
Lung	10	5.6
Liver and lung	1	0.5
No resection	141	79.7
**Treatment line of regorafenib**		
Third-line	114	64.4
Forth or late-line	63	35.6
**Previous biologics treatment^#^**		
anti-EGFR	65	36.7
anti-VEGF	138	77.9
**Starting dose(mg)**		
80	94	53.1
120	83	46.9
**Final dose(mg)**		
≤80	155	87.6
120	18	15.5

pMMR, proficient mismatch repair; dMMR, deficient mismatch repair.

*Right-sided included tumors from cecal to two thirds of proximal transverse colon; left-sided represented tumors from one third of distal transverse colon to rectum (not including rectum).

^†^According to the site of metastases, patients were divided into four groups: (1) liver limited MET; (2) lung limited MET; (3) liver and lung MET (4) other MET.

^#^Anti-EGFR, Cetuximab or panitumumab; Anti-VEGF, Bevacizumab.

Of the 177 patients, 116 (65.5%) received regorafenib alone (monotherapy group), while 28 (15.9%) and 33 (18.6%) were administered regorafenib in combination with chemotherapy (chemo group) and anti-PD1 antibodies (immune group), respectively (**Figure 1**). The median treatment line of all three groups was 3. The baseline data of each cohort are shown in **Table 2**.

**Table 2:** Baseline demographic and clinical characteristics of 3 study cohorts.

Characteristics	Monotherapy group (n = 116)		Chemo group (n = 28)		Immune group (n = 33)		p-value
	n	%	n	%	n	%	
**Sex**							0.234
Men	62	53.4	17	60.7	23	69.7	
Women	54	46.6	11	39.3	10	30.3	
**Age(years)**							0.135
Median (range)	61 (32–82)		60.5 (36–71)		51 (33–71)		
<65	74	63.8	20	71.4	27	81.8	
≥65	42	36.2	8	28.6	6	18.2	
Site of primary disease*							**0.015**
Left-sided	47	40.5	12	42.9	23	69.7	
Right-sided	22	19	8	28.6	6	18.2	
Rectum	47	40.5	8	28.6	4	12.1	
**Gene mutation status**							0.665
RAS/BRAF wild-type	58	50.0	12	42.9	14	42.4	
RAS mutant	57	49.1	14	50.0	17	51.5	
BRAF mutant	2	1.7	1	3.6	2	6.1	
**MMR status**							0.658
pMMR	114	98.3	28	100	32	97.0	
dMMR	2	1.7	0	0	1	3.0	
**Site of metastases^†^**							0.798
Lung limited	22	19.0	5	17.9	8	24.2	
Liver limited	53	45.7	14	50.0	10	30.3	
Liver and lung	23	19.8	5	17.9	8	24.2	
Other	18	15.5	4	14.3	7	21.2	
**Primary tumor resection**							0.652
Yes	99	85.3	25	89.3	30	90.9	
No	17	14.7	3	10.7	3	9.1	
**Treatment line of regorafenib**							0.148
Third-line	76	65.5	21	75	17	51.5	
Forth or late-line	40	34.5	7	25	16	48.5	
**Previous biologics treatment^#^**							
anti-EGFR	42	36.2	11	39.3	12	36.4	0.954
anti-VEGF	92	79.3	20	71.4	26	78.8	0.660
**Starting dose(mg)**							0.901
80	63	54.3	14	50.0	17	51.5	
120	53	45.7	14	50.0	16	48.5	
**Final dose(mg)**							0.460
≤80	104	81.9	24	85.7	27	78.8	
120	12	10.3	4	14.3	6	18.2	

pMMR, proficient mismatch repair; dMMR, deficient mismatch repair.

Bold values indicate p < 0.05.

*Right-sided included tumors from cecal to two thirds of proximal transverse colon; left-sided represented tumors from one third of distal transverse colon to rectum (not including rectum).

^†^According to the site of metastases, patients were divided into four groups: (1) liver limited MET; (2) lung limited MET; (3) liver and lung MET (4) other MET.

^#^Anti-EGFR, Cetuximab or panitumumab; Anti-VEGF, Bevacizumab.

### Efficacy and Survival Analysis

**Figure 2:**
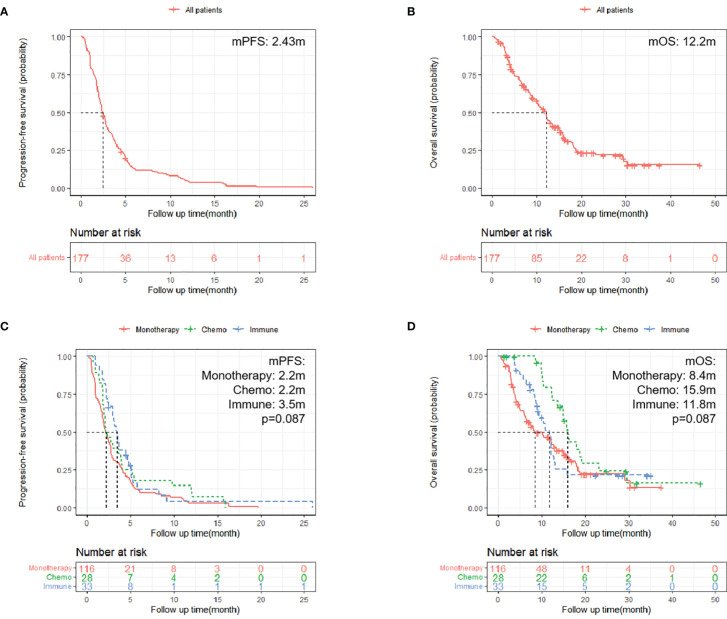
Kaplan–Meier survival curves. **(A, B)** Kaplan–Meier survival curves of all patients on PFS and OS [**(A)** PFS of all 177 patients; **(B)** OS of all 177 patients]; **(C, D)** Kaplan–Meier survival curves of different treatment patterns [**(C)** PFS of patients in three groups, p = 0.087; **(D)** OS in patients in three groups, p = 0.087].

In the 177 patients, no patient had a complete response. Only one patient had a partial response, indicating an objective response rate of 0.56%. Disease control was achieved in 72 (40.7%) of the 177 patients. Regorafenib showed an mPFS of 2.43 months (95% CI: 2.17–2.83) and an mOS of 12.2 months (95% CI: 10.2–13.6) (**Figures 2A, B**).

PFS in the immune group was significantly better than that in the monotherapy group (3.5 m vs. 2.2 m, HR = 0.65; 95% CI: 0.43–0.99; p = 0.043; **Figure 2C** and **Supplementary Figure 1A**). However, OS did not reach significance (11.8 m vs. 8.4 m; p = 0.37; **Supplementary Figure 1B**). Although PFS was not significantly longer in the chemo group compared with the monotherapy group (2.2 m vs. 2.2 m; p = 0.25; **Supplementary Figure 1C**), OS in the chemo group was significantly longer than that in the monotherapy group (15.9 m vs. 8.4 m, HR = 0.57; 95% CI: 0.34–0.95; p = 0.032; **Figure 2D** and **Supplementary Figure 1D**).

In the chemo group, chemo regimens included single regimen (irinotecan, capecitabine or raltitrexed) and doublet regimen (FOLFOX, irinotecan plus raltitrexed or oxaliplatin plus raltitrexed). Detailed features of these regimens are listed in **Supplementary Table 1**. There were no significant differences in PFS (2.0 m vs. 2.7 m; p = 0.11) and OS (14.9 m vs. 18.1 m; p = 0.52) between the single and doublet chemotherapy groups (**Supplementary Figures 2A, B**).

Based on the site of metastasis (MET), the patients were divided into four groups: (1) liver limited MET; (2) lung limited MET; (3) liver and lung MET; and (4) other MET. Patients with lung limited MET had the longest PFS compared with liver limited MET, liver and lung MET, and other MET but there was no significant difference (mPFS: 2.9 m vs. 2.2 m, 2.3 m and 2.2 m; p = 0.054; **Supplementary Figure 2C**).

**Figure 3:**
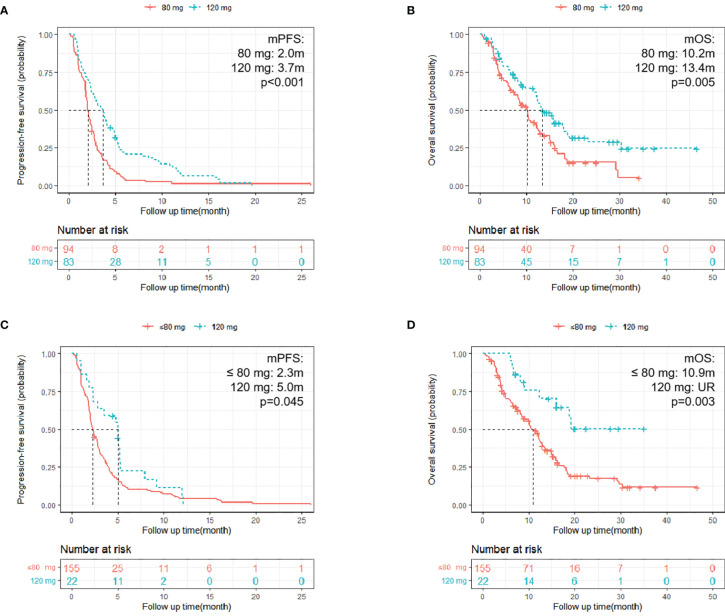
Kaplan–Meier survival curves. **(A, B)** Kaplan–Meier survival curves of different starting doses on PFS and OS [**(A)** PFS of patients with different start doses, p < 0.001; **(B)** OS of patients with different starting doses; p = 0.005]; **(C, D)** Kaplan–Meier survival curves of different final doses on PFS and OS [**(C)** PFS of patients with different final doses, p = 0.045; **(D)** OS of patients with different final doses; p = 0.003].

A total of 94 (53.1%) patients had a starting dose of 80 mg, and 83 (46.9%) patients began treatment at 120 mg, among whom 62 (74.7%) had dose modifications. Only one patient began at 80 mg with a final dose of 120 mg. Finally, 155 patients (87.5%) had a final dose of 80 mg or less than 80 mg, and 22 (12.4%) patients had final doses of 120 mg. Patients with a starting dose of 120 mg had longer PFS and OS compared with those who began treatment at 80 mg (PFS: mPFS: 3.7 m vs. 2.0 m; HR = 0.52; 95% CI: 0.38–0.71; p <0.001; **Figure 3A**; OS: mOS: 13.4 m vs. 10.2 m; HR = 0.59; 95% CI: 0.41–0.86; p = 0.005; **Figure 3B**). Furthermore, patients with a final dose of 120 mg had longer PFS compared with the 80 mg or less groups (PFS: HR = 0.61; 95% CI: 0.38–0.99; p = 0.045; mPFS: 5.0 m vs. 2.3 m; **Figure 3C**; OS: HR = 0.35; 95% CI: 0.18–0.70; p = 0.003; mOS: UR vs. 10.9 m; **Figure 3D**).

Univariable and multivariable COX regression analyses were performed. The results showed that primary tumor resection, a starting dose of 120 mg and lung limited metastatic disease were independent prognostic factors of PFS (**Supplementary Table 2**). Primary tumor resection and a final dose of 120 mg were independent prognostic factors of OS (**Supplementary Table 3**).

### Adverse Effects

All 177 patients were assessed for toxicity. The rate of any grade AEs was 87.0% (154/177). Common treatment-related adverse events (TRAEs) of any grade included hand-foot skin reaction (71 [25.9%]), fatigue (39 [22.0%]), elevated liver enzymes (35 [19.8%]) and decreased appetite (18 [10.1%]). The rate of grade 3 or 4 toxicity was 42.4% (75/177), including hand-foot skin reaction (n = 25), elevated liver enzymes (n = 13), fatigue (n = 7), leucopenia (n = 6), thrombocytopenia (n = 6), hypertension (n = 5), rash (n = 3), decreased appetite (n = 2), proteinuria (n = 2), ileus (n = 2), hoarseness (n = 2), oral mucositis (n = 1), and diarrhea (n = 1). No treatment-related death occurred.

The rate of any AEs in the monotherapy group, the chemo group and the immune group were 86.2, 89.3, and 87.9%, respectively (p = 0.89). The rate of grade ≥3 AEs in three groups were 41.3, 46.4, and 42.4%, respectively (p = 0.88). There was no significant difference among three groups in terms of AEs. The detailed AEs are listed in **Table 3**.

**Table 3:** Adverse events of regorafenib treatment.

	Monotherapy group (n = 116)			Chemo group (n = 28)			Immune group (n = 33)			p-value^#^
	Any grade	Grade1–2	Grade ≥3	Any grade	Grade1–2	Grade ≥3	Any grade	Grade1–2	Grade ≥3	
Any event*	100 (86.2%)	52 (44.8%)	48 (41.3%)	25 (89.3%)	12 (42.8%)	13 (46.4%)	29 (87.9%)	15 (45.4%)	14 (42.4%)	0.89
Hand–foot skin reaction	45 (38.8%)	29 (25%)	16 (13.8%)	13 (46.4%)	8 (28.6%)	5 (17.8%)	13 (39.4%)	9 (27.3%)	4 (12.1%)	0.75
Rash	6 (5.2%)	5 (4.3%)	1 (0.8%)	2 (7.1%)	1 (3.5%)	1 (3.5%)	1 (3%)	0 (0)	1 (3%)	0.76
Oral mucositis	4 (3.4%)	3 (2.6%)	1 (0.8%)	1 (3.5%)	1 (3.5%)	0 (0)	0 (0)	0 (0)	0 (0)	0.55
Fatigue	23 (19.8%)	18 (15.5%)	5 (4.3%)	6 (21.4%)	6 (21.4%)	0 (0)	10 (30.3%)	8 (24.2%)	2 (6.1%)	0.43
Hypertension	11 (9.5%)	7 (6%)	4 (3.4%)	1 (3.6%)	1 (3.5%)	0 (0)	6 (18.2%)	5 (15.2%)	1 (3%)	0.15
Proteinuria	8 (6.9%)	6 (5.2%)	2 (1.7%)	2 (7.1%)	2 (7.1%)	0 (0)	1 (3%)	1 (3%)	0 (0)	0.70
Elevated liver enzymes	22 (18.9%)	15 (12.9%)	7 (6%)	6 (21.4%)	3 (10.7%)	3 (10.7%)	7 (21.2%)	4 (12.1%)	3 (9.1%)	0.93
Diarrhea	3 (2.6%)	2 (1.7%)	1 (0.8%)	1 (3.5%)	1 (3.5%)	0 (0)	0 (0)	0 (0)	0 (0)	0.59
Decreased appetite	12 (10.3%)	10 (8.6%)	2 (1.7%)	3 (10.7%)	3 (10.7%)	0 (0)	3 (9.1%)	3 (9.1%)	0 (0)	0.97
Leucopenia	10 (8.6%)	7 (6%)	3 (2.6%)	5 (17.8%)	3 (10.7%)	2 (7.1%)	2 (6.1%)	1 (3%)	1 (3%)	0.24
Thrombocytopenia	8 (6.9%)	5 (4.3%)	3 (2.6%)	5 (17.8%)	3 (10.7%)	2 (7.1%)	2 (6.1%)	1 (3%)	1 (3%)	0.14
Other^†^	5 (4.3%)	2 (1.7%)	3 (2.6%)	0 (0)	0 (0)	0 (0)	4 (12.1%)	3 (9.1%)	1(3%)	0.08

Data presented as No. (%).

*For patients with more than one adverse event, only the highest grade of the most severe event is shown.

^†^Others include ileus (n = 2), hoarseness (n = 2) and myalgia (n = 1) in the monotherapy group; hypothyroidism (n = 1), hoarseness (n = 1), lipase elevate (n = 1) and myocardial enzyme elevation (n = 1) in the immune group.

^#^p-value was calculated using any grade of AEs.

## Discussion

This multi-center study represented the real-world fact of regorafenib in the treatment of refractory mCRC. This study firstly comparatively assessed the efficacy and safety among three cohorts (regorafenib alone, regorafenib plus chemotherapy and regorafenib plus anti-PD1 antibodies), and data were collected from real-world practice based on multi-disciplinary team (MDT) mode.

As standard salvage treatment, regorafenib has limited survival benefit, leading to a series of trials exploring the possibility of its combination with other agents, including immunotherapeutic drugs. The median PFS in the immune group of our cohort was 3.5 months, it was much shorter than the REGONIVO trial (mPFS: 7.9 months) (17), but consistent with other trials including the REGOMUNE trial (mPFS: 3.6 months) (18), the REGOTORI trial (mPFS: 2.6 months) and some small-size retrospective studies (19–21). A possible reason is that the ROGONIVO study recruited a higher proportion of patients with lung metastasis than the present trial (64% vs. 40%). These results suggest that although regorafenib plus immunotherapy may prolong survival, it is of great importance to determine the proper population and suitable biologics with the greatest benefit from this strategy.

Another strategy is to combine regorafenib with chemotherapy. In the current cohort, the superiority of this combination in PFS improvement was not observed (mPFS: 2.25 m vs. 2.18 m, p = 0.25), whereas the chemo group had better OS (mOS: 15.9 m vs. 8.4 m, p = 0.032). A single-arm retrospective study included 41 patients administered regorafenib combined with FOLFIRI with dose-escalated irinotecan, where an overall DCR of 58.5% was determined, and the median PFS and OS were 6.0 and 12.0 months, respectively (22). In another small-size retrospective study, regorafenib plus chemo had significantly higher PFS and OS than regorafenib monotherapy (mPFS: 3.7 m vs. 2.5 m, p = 0.009; mOS: 20.9 m vs. 10.3 m, p = 0.015) (23). The chemotherapy regimens in this study were determined by an MDT, varying from irinotecan alone to doublet treatments like FOLFOX. Patients in the real-world late-line setting have poor performance status, and it is unlikely for all to receive intense therapy like FOLFIRI. It is of great interest to determine the best regimen to combine with regorafenib. Our study showed no significant difference of survival between patients treated with regorafenib plus single chemotherapeutic agent and those administered doublet therapies. Due to the small size of our chemo cohort, we failed to analyze each regimen, which may deserve further investigation.

As shown above, patients with lung limited MET had the longest PFS, corroborating a previous retrospective study, indicating the presence of a lung limited metastatic disease is significantly associated with better clinical outcome in patients with mCRC administered regorafenib (27). We found patients who began treatment at 120 mg had significantly longer PFS and OS compared with those who began at 80 mg. Patients with a final dose of 120 mg had the longer PFS and OS compared with those with a final dose ≤80 mg. It could be understood that patients had higher doses had higher plasma drug concentration and tended to had better physical performance. In terms of clinical outcome, data of the present study was similar to those of other studies in which most patients began regorafenib treatment at 160 mg, and the present data indicated that 120 mg might be more suitable for Asian patients.

Toxicity is a key problem in real-world clinical practice. This study found that the rate of any grade AEs was 87.0% and the rate of grade ≥3 AEs was 42.4%. In the CORRECT study (10), 93% patients had any grade AEs and up to 54% patients had grade ≥3 AEs. It could be explained that patients in the CORRECT study were heavily treated before regorafenib treatment (49% patients had ≥4 previous systemic therapies), while 64.4% patients of our cohort received only two prior line of therapy. And most patients in the CORRECT study started at 160 mg whereas most patients in this study had a starting dose of 120 mg. We found combined strategy did not significantly increase the rate of AEs since the rate of AEs in three groups were similar (86.2% vs. 89.3% vs. 87.9%; p = 0.89). Furthermore, patients administered regorafenib alone and in combination with other agents showed different AEs. Patients in the chemo group had highest rate of leucopenia (17.8%), while patients in the immune group had highest rates of hypertension (18.2%).

This study had limitations and bias inherent to observational retrospective studies. The retrospective and non-randomized nature of this study makes it subjective to certain selection bias. In addition, ECOG PS (the Eastern Cooperative Oncology Group performance status) data were not available for most patients.

In summary, we have provided real-world data for regorafenib treatment in four colorectal centers in China. We found patients administered regorafenib plus anti-PD1 antibodies had longer PFS than those treated with regorafenib alone or combined with chemotherapy, while the regorafenib plus chemotherapy group had longer OS. These findings require further confirmation by prospective studies.

## Data Availability Statement

The raw data supporting the conclusions of this article will be made available by the authors, without undue reservation.

## Ethics Statement

The studies involving human participants were reviewed and approved by the Ethics Review Board of Zhongshan Hospital of Fudan University, the Ethics Review Board of the First Affiliated Hospital of Wenzhou Medical University, the Ethics Review Board of the Third Affiliated Hospital of Soochow University, and the Ethics Review Board of Qilu Hospital of Shandong University. The patients/participants provided their written informed consent to participate in this study.

## Author Contributions

WJC, JMX, LCY, JMZ, and JSL designed the project. DHX, YL, WTT, and LSX collected information of the patients and wrote the manuscript. TYL, YDJ, SZZ, and XRQ helped with the data analysis. All authors listed have made a substantial, direct, and intellectual contribution to the work and approved it for publication.

## Funding

This work was funded by the National Natural Science Foundation of China (82072653, 81602035, 82072678, 82172816); the Fujian Province Health Care Foundation (2021GGB032); the Clinical research project of Health Professions of Shanghai Municipal Health Commission (20214Y0277); the Clinical Research Plan of SHDC (SHDC2020CR3037B, SHDC2020CR1033B, SHDC 2020CR5006); the Zhejiang Provincial Natural Science Foundation of China (LY22H160021) and the Shandong Provincial Natural Science Foundation (ZR2020LZL018).

## Conflict of Interest

The authors declare that the research was conducted in the absence of any commercial or financial relationships that could be construed as a potential conflict of interest.

## Publisher’s Note

All claims expressed in this article are solely those of the authors and do not necessarily represent those of their affiliated organizations, or those of the publisher, the editors and the reviewers. Any product that may be evaluated in this article, or claim that may be made by its manufacturer, is not guaranteed or endorsed by the publisher.

## Supplementary Material

Supplementary Figure 1 Kaplan–Meier survival curves. (**A**: PFS of patients in the immune group and monotherapy group, p = 0.043; **B**: OS of patients in the immune group and monotherapy group, p = 0.37; **C**: PFS of patients in the chemo group and monotherapy group, p = 0.25; **D**: OS of patients in the chemo group and monotherapy group, p = 0.032; **E**: PFS of patients in the chemo group and immune group, p=0.59; **F**: OS of patients in the chemo group and immune group, p = 0.074).
