# Supplementary material for: Regorafenib in Refractory Metastatic Colorectal Cancer: A Multi-Center Retrospective Study
Source: Front Oncol. 2022 Mar 30;12:838870. doi: 10.3389/fonc.2022.838870 (PMC9007238; doi:10.3389/fonc.2022.838870)
Supplement: Supplementary Figure 1 — Kaplan–Meier survival curves. (A: PFS of patients in the immune group and monotherapy group, p = 0.043; B: OS of patients in the immune group and monotherapy group, p = 0.37; C: PFS of patients in the chemo group and monotherapy group, p = 0.25; D: OS of patients in the chemo group and monotherapy group, p = 0.032; E: PFS of patients in the chemo group and immune group, p=0.59; F: OS of patients in the chemo group and immune group, p = 0.074). [file DataSheet_1.docx]

Supplementary Material

# Supplementary Figures

**
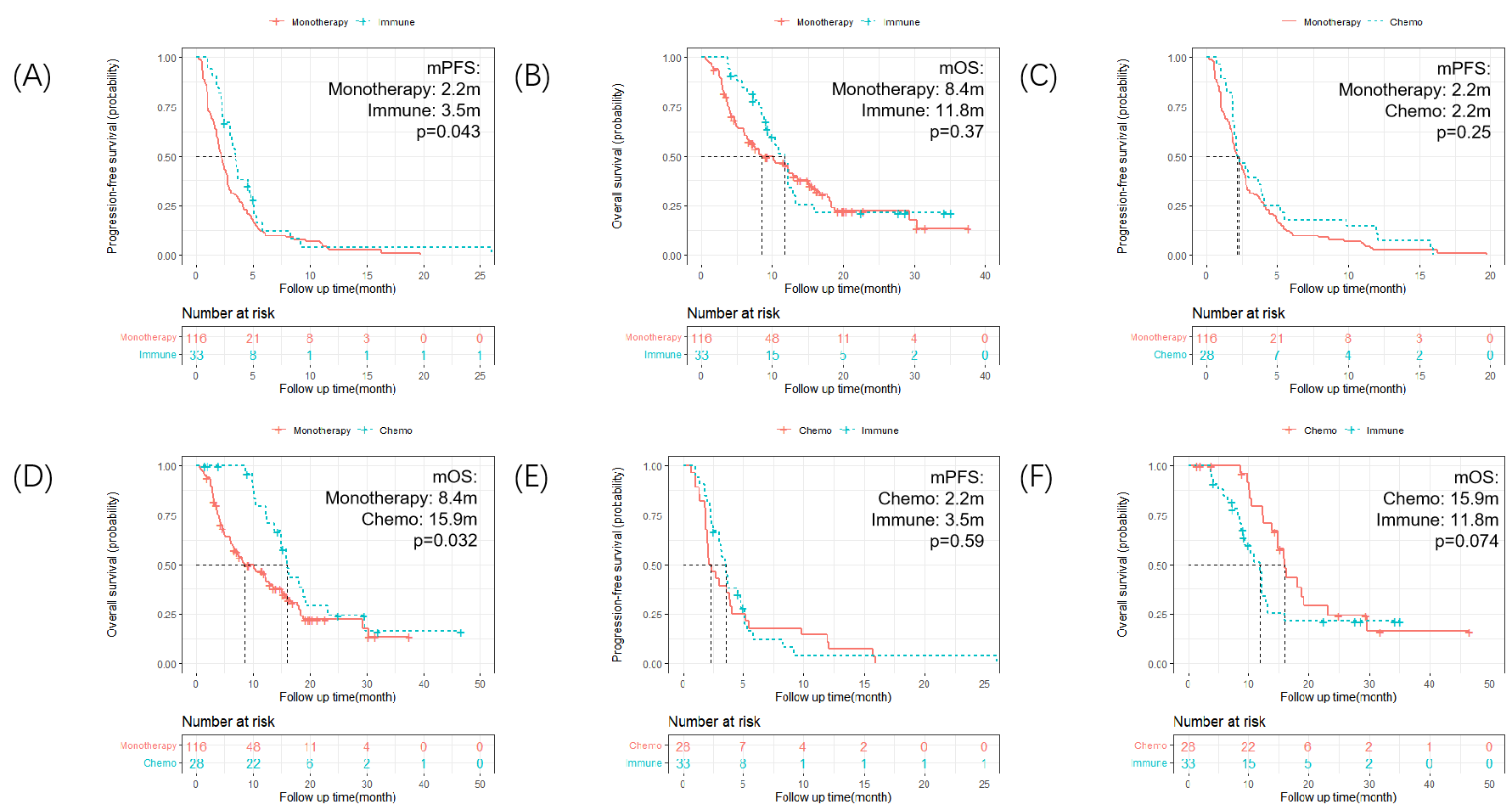
**

Supplementary Figure 1. Kaplan–Meier survival curves. (a: PFS of patients in the immune group and monotherapy group, p=0.043; b: OS of patients in the immune group and monotherapy group, p=0.37; c: PFS of patients in the chemo group and monotherapy group, p=0.25; d: OS of patients in the chemo group and monotherapy group, p=0.032; e: PFS of patients in the chemo group and immune group, p=0.59; f: OS of patients in the chemo group and immnue group, p=0.074).

**
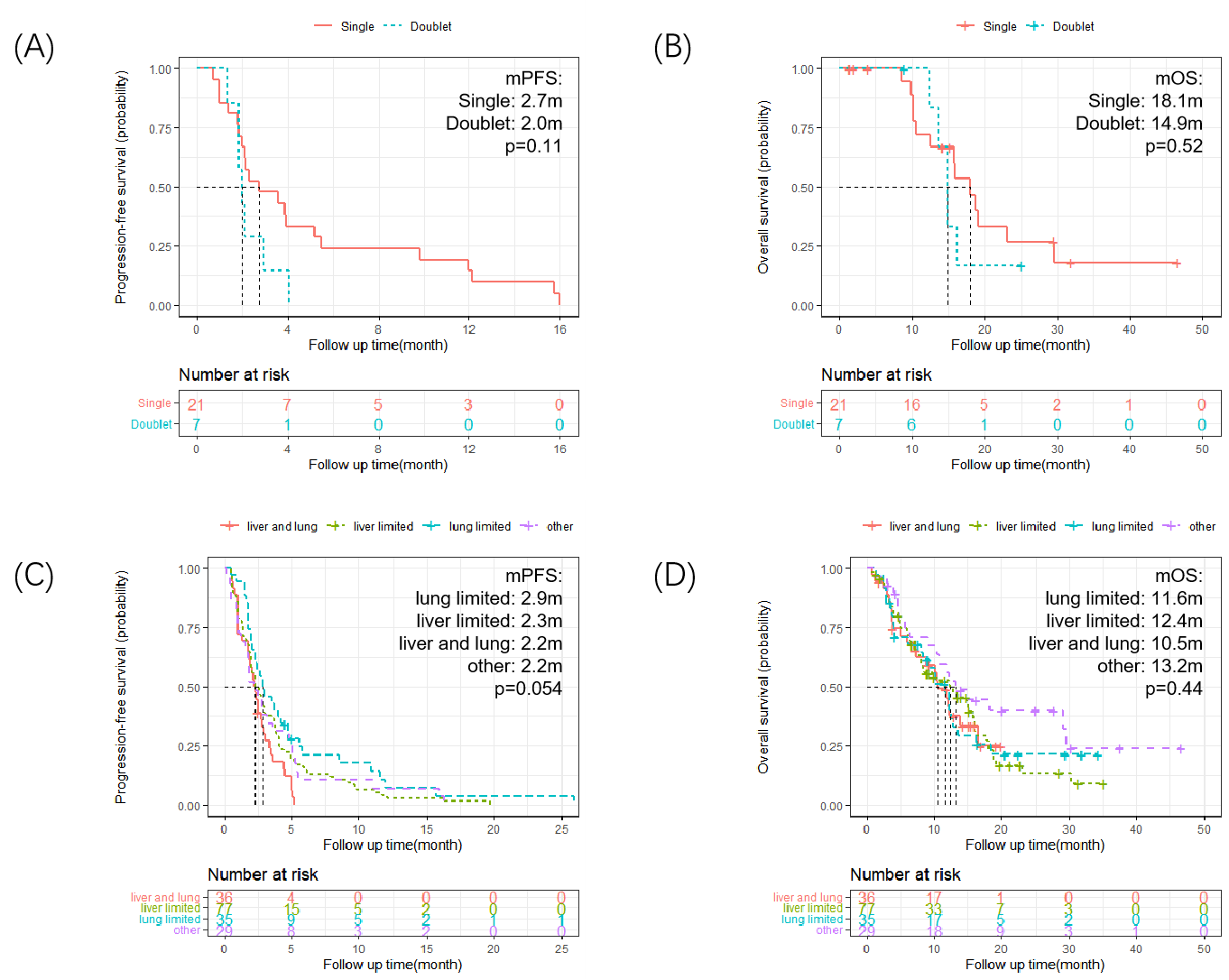
**

Supplementary Figure 2. Kaplan–Meier survival curves. a-b: Kaplan–Meier survival curves of different chemotherapy regimes in chemo group (a: PFS of patients in chemo group, p=0.11; b: OS of patients in chemo group, p=0.52)；c-d: Kaplan–Meier survival curves of different sites of metastases (c: PFS of patients with different sites of metastases, p=0.054; d: OS of patients with different sites of metastases, p=0.44).

# Supplement Tables

Supplement table1: Chemotherapy regimens of chemo group.

| Chemotherapy regimes | Chemo group(n=28) | |
| --- | --- | --- |
|  | n | % |
| Total | 28 | 100 |
| Capecitabine | 11 | 39.3 |
| Irinotecan | 2 | 7.1 |
| Raltitrexed | 8 | 28.6 |
| FOLFOX* | 3 | 10.7 |
| Oxaliplatin plus raltitrexed | 2 | 7.1 |
| Irinotecan plus raltitrexed | 2 | 7.1 |

*FOLFOX, leucovorin, fluorouracil, and oxaliplatin.

Supplement table 2: Univariate and multivariate analysis of PFS.

|  | Univariate analysis | | Multivariate analysis | |
| --- | --- | --- | --- | --- |
|  | HR (95% CI) | p value | HR (95% CI) | p value |
| **Sex** |  |  |  |  |
| Women | Reference |  |  |  |
| Men | 0.77(0.57-1.1) | 0.1 |  |  |
| **Age** |  |  |  |  |
| <65 | Reference |  |  |  |
| ≥65 | 0.83(0.6-1.1) | 0.26 |  |  |
| **Site of primary disease*** |  |  |  |  |
| Left-sided | Reference |  |  |  |
| Right-sided | 1.08(0.72-1.61) | 0.69 |  |  |
| Rectum | 0.82(0.58-1.16) | 0.27 |  |  |
| **Gene mutation status** |  |  |  |  |
| RAS/BRAF wild-type | Reference |  |  |  |
| RAS mutant | 0.83(0.61-1.13) | 0.24 |  |  |
| BRAF mutant | 1.35(0.54-3.37) | 0.5 |  |  |
| **MMR status** |  |  |  |  |
| pMMR | Reference |  |  |  |
| dMMR | 0.81(0.25-2.57) | 0.72 |  |  |
| **Site of metastases**† |  |  |  |  |
| Liver and lung | Reference |  | Reference |  |
| Liver limited | 0.68(0.45-1.03) | 0.07 | 0.69(0.46-1) | 0.07 |
| Lung limited | 0.50(0.30-0.82) | **0.006** | 0.51(0.31-0.84) | **0.007** |
| Other | 0.68(0.41-1.13) | 0.14 | 0.7(0.42-1.2) | 0.17 |
| **Primary tumor resection** |  |  |  |  |
| No | Reference |  | Reference |  |
| Yes | 0.48(0.3-0.75) | **0.0015** | 0.56(0.35-0.89) | **0.01** |
| **Treatment line of regorafenib** |  |  |  |  |
| Third-line | Reference |  |  |  |
| Forth or late-line | 0.94(0.68-1.3) | 0.68 |  |  |
| **Previous anti-EGFR#** |  |  |  |  |
| No | Reference |  |  |  |
| Yes | 0.98(0.72-1.3) | 0.89 |  |  |
| **Previous anti-VEGF^** |  |  |  |  |
| No | Reference |  |  |  |
| Yes | 1.1(0.79-1.6) | 0.5 |  |  |
| **Starting dose(mg)** |  |  |  |  |
| 80 | Reference |  | Reference |  |
| 120 | 0.52(0.38-0.71) | **<0.001** | 0.57(0.4-0.79) | **<0.001** |
| **Final dose(mg)** |  |  |  |  |
| ≤80 | Reference |  | Reference |  |
| 120 | 0.61(0.38-0.99) | **0.04** | 0.8(0.48-1.3) | 0.4 |

Bold values indicate p < 0.05.

PFS, progression-free survival.

*Right-sided included tumors from cecal to two thirds of proximal transverse colon; left-sided represented tumors from one third of distal

transverse colon to rectum (not including rectum).

†According to the site of metastases, patients were divided into four groups: (1) liver limited MET; (2) lung limited MET; (3) liver and lung MET (4) other MET.

#Anti-EGFR:Cetuximab or panitumumab.

^Anti-VEGF: Bevacizumab.

Supplement table 3: Univariate and multivariate analysis of OS.

|  | Univariate analysis | | Multivariate analysis | |
| --- | --- | --- | --- | --- |
|  | HR (95% CI) | p value | HR (95% CI) | p value |
| **Sex** |  |  |  |  |
| Women | Reference |  |  |  |
| Men | 0.79 (0.54-1.14) | 0.2 |  |  |
| **Age** |  |  |  |  |
| <65 | Reference |  |  |  |
| ≥65 | 0.(0.6-1.1) | 0.88 |  |  |
| **Site of primary disease*** |  |  |  |  |
| Left-sided | Reference |  |  |  |
| Right-sided | 1.06(0.65-1.72) | 0.81 |  |  |
| Rectum | 0.88(0.58-1.33) | 0.55 |  |  |
| **Gene mutation status** |  |  |  |  |
| RAS/BRAF wild-type | Reference |  |  |  |
| RAS mutant | 1.09(0.75-1.59) | 0.61 |  |  |
| BRAF mutant | 1.59(0.63-3.99) | 0.31 |  |  |
| **MMR status** |  |  |  |  |
| pMMR | Reference |  |  |  |
| dMMR | 0.66(0.09-4.78) | 0.68 |  |  |
| **Site of metastases**† |  |  |  |  |
| Liver and lung | Reference |  |  |  |
| Liver limited | 0.97(0.59-1.61) | 0.93 |  |  |
| Lung limited | 0.95(0.53-1.71) | 0.88 |  |  |
| Other | 0.64(0.34-1.2) | 0.17 |  |  |
| **Primary tumor resection** |  |  |  |  |
| No | Reference |  | Reference |  |
| Yes | 0.48(0.29-0.79) | **0.004** | 0.56(0.34-0.93) | **0.02** |
| **Treatment line of regorafenib** |  |  |  |  |
| Third-line | Reference |  |  |  |
| Forth or late-line | 1.35(0.93-1.96) | 0.1 |  |  |
| **Previous anti-EGFR#** |  |  |  |  |
| No | Reference |  |  |  |
| Yes | 0.93(0.63-1.36) | 0.71 |  |  |
| **Previous anti-VEGF^** |  |  |  |  |
| No | Reference |  |  |  |
| Yes | 1.31(0.85-2.03) | 0.21 |  |  |
| **Starting dose(mg)** |  |  |  |  |
| 80 | Reference |  | Reference |  |
| 120 | 0.59(0.41-0.86) | **0.005** | 0.72(0.49-1.07) | 0.1 |
| **Final dose(mg)** |  |  |  |  |
| ≤80 | Reference |  | Reference |  |
| 120 | 0.35(0.18-0.7) | **0.003** | 0.44(0.21-0.91) | **0.02** |

Bold values indicate p < 0.05.

PFS, progression-free survival.

*Right-sided included tumors from cecal to two thirds of proximal transverse colon; left-sided represented tumors from one third of distal

transverse colon to rectum (not including rectum).

†According to the site of metastases, patients were divided into four groups: (1) liver limited MET; (2) lung limited MET; (3) liver and lung MET (4) other MET.

#Anti-EGFR:Cetuximab or panitumumab.

^Anti-VEGF: Bevacizumab.
